# POOR GLUCOSE REGULATION IS ASSOCIATED WITH LOWER WELL-BEING AMONG OLDER MEN, BUT NOT WOMEN

**DOI:** 10.1093/geroni/igz038.1626

**Published:** 2019-11-08

**Authors:** Konstantinos Mantantzis, Johanna Drewelies, Gert G Wagner, Ilja Demuth, Elizabeth Steinhagen-Thiessen, Ulman Lindenberger, Sandra Düzel, Denis Gerstorf

**Affiliations:** 1 Humboldt University of Berlin, Berlin, Germany; 2 Humboldt University of Berlin, Berlin, Berlin, Germany; 3 German Institute for Economic Research, Berlin, Berlin, Germany; 4 Charité University Hospital, berlin, Berlin, Germany; 5 Max Planck Institute for Human Development, berlin, Berlin, Germany

## Abstract

Glucose regulation is a key aspect of healthy aging, but little is known about gluco-regulatory capacity and older adults’ well-being. In this study, we examine whether gluco-regulatory capacity is predictive of within-person age-related trajectories of three major well-being indicators. We applied growth models to multi-year longitudinal data obtained in the Berlin Aging Study II (N = 1437; age 60-89; 53% women) and used insulin resistance as an index of glucose regulation capacity. Poor glucose regulation was associated with lower levels of well-being in men, but not women. These associations among men emerged for two of the three well-being indicators, were maintained across old age, and were independent of the other cognitive and physical factors examined. We discuss how sexual dimorphism may have contributed to our findings, and conclude that our results provide initial evidence for the relevance of glucose regulation for quality of life among older men.

